# How to Measure the Aorta Using MRI: A Practical Guide

**DOI:** 10.1002/jmri.27183

**Published:** 2020-05-06

**Authors:** Max J. van Hout, Arthur J. Scholte, Joe F. Juffermans, Jos J. Westenberg, Liang Zhong, Xuhui Zhou, Simon M. Schalla, Michael D. Hope, Jens Bremerich, Christopher M. Kramer, Marc Dewey, Karen G. Ordovas, David A. Bluemke, Hildo J. Lamb

**Affiliations:** ^1^ Department of Cardiology Leiden University Medical Center Leiden The Netherlands; ^2^ Department of Radiology Leiden University Medical Center Leiden The Netherlands; ^3^ National Heart Centre Singapore National Heart Research Institute Singapore Singapore Singapore; ^4^ Cardiovascular Sciences Academic Clinical Programme, Duke‐NUS Medical School Singapore Singapore; ^5^ Department of Radiology Eighth Affiliated Hospital of Sun Yat‐sen University Shenzhen China; ^6^ Department of Cardiology Maastricht University Medical Centre Maastricht The Netherlands; ^7^ Department of Radiology University of California San Francisco California USA; ^8^ Department of Radiology Universitätsspital Basel Basel Switzerland; ^9^ Cardiovascular Division, Department of Medicine University of Virginia Charlottesville Virginia USA; ^10^ Department of Radiology Charité – Universitätsmedizin Berlin Berlin Germany; ^11^ Department of Radiology University of Wisconsin Madison Wisconsin USA

AORTIC DIMENSIONS ARE IMPORTANT in the risk assessment of aortic pathology, aneurysms, dissection, and rupture. However, there is much debate on how, when, and where to measure the aorta. The main imaging techniques used to measure the aorta are transthoracic echocardiography (TTE), computed tomography (CT), and magnetic resonance imaging (MRI). MRI has the advantage over TTE and CT in that it is able to accurately visualize the entire aorta without using ionizing radiation and is able to give additional information on ventricular, valvular and vascular function and flow dynamics. Proximal aortic diameter measurements can vary up to 5 mm between imaging modalities, which can lead to relevant differences in clinical decisions about preventive surgery.^1^ The American and European guidelines give recommendations on how to measure the aorta, but these recommendations differ and can be ambiguous.^2^, ^3^ There are limited MRI guidelines on how to measure the aorta.^1^, ^4^ Accordingly, still large variations exist in image acquisition and analysis in MRI.^5^ The question is: how, when, and where do the guidelines advise us to measure the aorta? We will discuss aortic analysis in MRI and compare these to accepted practice in CT and TTE. This article provides recommendations for clinicians on aortic measurements in the adult population using MRI, with an emphasis on the thoracic aorta.

## How, When, and Where to Measure

### *How: With or Without Aortic Vessel Wall?*

**FIGURE 1:**
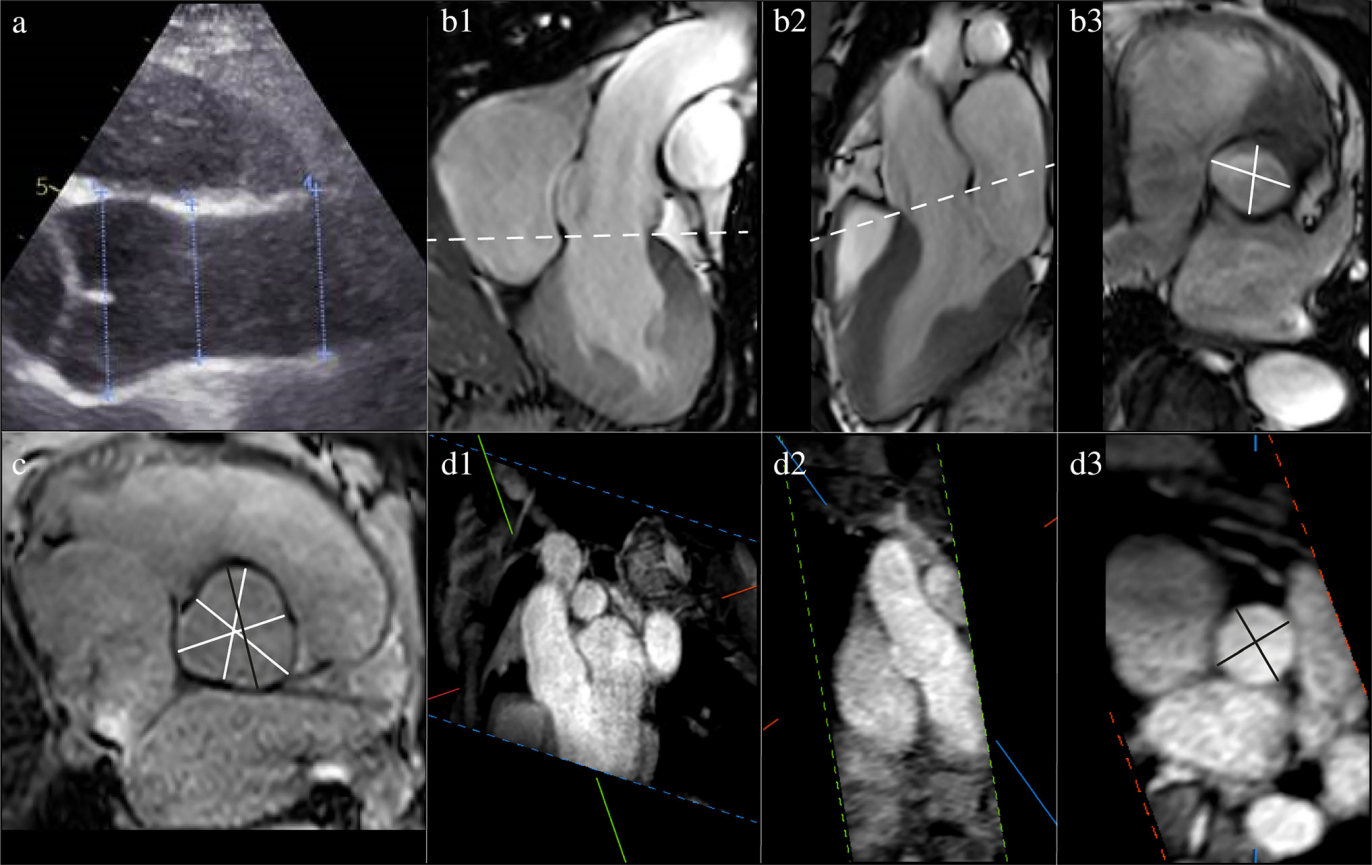
Echocardiographic and MRI measurements of a healthy proximal aorta. (**a**) Echocardiographic end‐diastolic leading edge‐to‐leading edge measurement of the sinus of Valsalva, sinotubular junction, and ascending aorta. (**b**) Coronal (b1) and sagittal (b2) planning views for double‐oblique (b3) MRI inner edge‐to‐inner edge systolic measurement of the annulus. (**c**) MRI average cusp‐to‐commissure and largest cusp‐to‐cusp measurement of the sinus during end‐diastole (with closed aortic valves). (**d**) MRA planning views (d1) and (d2) for double‐oblique (d3) MRI inner edge‐to‐inner edge diastolic measurement of the sinotubular junction.

**FIGURE 2:**
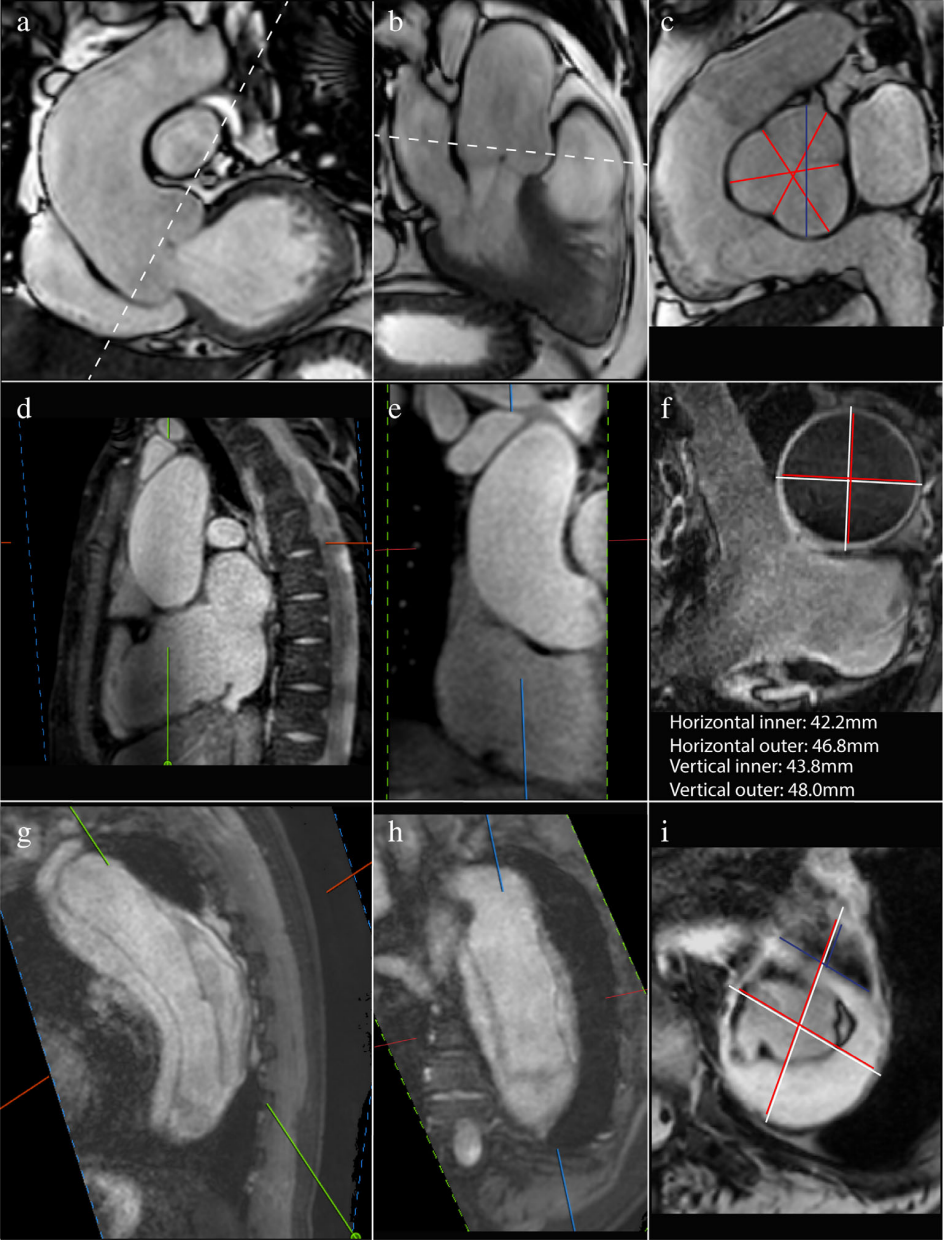
MRI images of pathologic aorta's. Top three images: dilated sinus planned on (**a**) coronal and (**b**) sagittal views for (**c**) double‐oblique I‐I measurement (SSFP cine only provides luminal enhancement, vessel wall not clearly deliniated): average cusp‐to‐commissure (in red) and largest cusp‐to‐cusp diameter (in blue). Three middle images: bicuspid valve with dilated ascending aorta with MRA planning views (**d**) and (**e,f**) Black‐blood double‐oblique measurement (red: I‐I, white: O‐O). Three images below: type B aortic dissection with mural thrombus, MRA planning views (**g–i**) Black‐blood double‐oblique measurement (red: I‐I, white: O‐O, blue: true lumen, crossing of red and white lines: false lumen surrounded by mural thrombus).

The normal ascending aortic vessel wall is ~2 mm thick, so inclusion of the wall can account for a 4‐mm difference in aortic size.^6^ Echocardiography guidelines recommend the leading edge‐to‐leading edge (L‐L) method (Fig. 1a) for measuring aortic diameters; consequently, the L‐L method has been used in many trials that defined normal aortic size limits.^1^ Studies comparing multimodality imaging techniques have shown that the inner edge‐to‐inner edge (I‐I) method (Fig. 1b–d) for MRI is in best agreement with the L‐L method of echocardiography.^1^ Given the high conformity with echocardiographic measurements and that with bright‐blood MR angiography (MRA) techniques, only the equivalent of I‐I measurements are obtained, and it would be advisable to use the I‐I method in MRI. In the case of wall thickening or aneurysm formation using the I‐I method, the external diameter should also be reported, as it aids in surgical or transarterial intervention planning (Fig. 2a–f).^4^, ^7^ For the outer–outer measurements, black‐blood images are required.

For image acquisition, MRI provides multiple possibilities to obtain luminal enhancement. Gadolinium contrast‐enhanced images can provide high‐resolution images of aortic lumen; however, when possible it is advisable to avoid application of contrast medium.^2^ If prolonged imaging is required noncontrast‐enhanced imaging techniques can be used, such as: black‐blood (using fast spin‐echo), bright‐blood (using time‐of‐flight, phase‐contrast or steady‐state free‐precession [SSFP]), water‐fat separated 3D‐imaging or 4D‐flow imaging.^1^, ^8^ It is advisable to use the same acquisition technique during follow‐up to aid measurement consistency. Table 1 provides an overview of recommendations for hardware as well as main sequence characteristics.

**TABLE 1:** Noncontrast‐Enhanced MRA Imaging Parameters

*Group*	Parameter	Ideal situation	Limiting factor	Recommendation
*Hardware*	Field strength:	1.5 or 3T	Availability. 3T provides higher SNR with also higher susceptibility for metallic artefacts.	Either 1.5 or 3T
	Number of coil elements:	Maximum, for optimal SNR.	Availability.	Maximum available.
*General sequence characteristics*	Field of view:	Maximum, for optimal SNR and coverage.	Scan time, magnetic field inhomogeneity.	Cover region of interest, 3D for double‐oblique reformatting.
	Spatial resolution:	Maximum, for optimal accuracy. Isotropic.	Scan time, SNR.	In‐plane voxel size of <1.5 × 1.5 mm^2^.
	Temporal resolution:	Cine images: Maximum, for optimal accuracy.	Scan time.	Cine Images: <40 msec/ heart phase.
	ECG synchronization:	‐ Cine images: retrospective, for coverage of the entire cardiac cycle.	‐ Cine images: Reconstruction complexity.	‐ Cine images: if available retrospective, otherwise prospective.
				‐ Bright & black blood: gating at 600–1200 msec, depending on heart rate.
			‐ Bright & black blood: Heart rate variation.	
		‐ Bright & black blood: Prospective, triggered at end‐diastole.		
	Respiratory motion compensation:	Use motion correction for optimal accuracy.	Scan time, breathing artefacts, reconstruction complexity.	Diameter measurement of the aortic root, ascending aorta, aortic arch and thoracic descending aorta: Respiration motion compensation using: self‐navigation, bellows gating, gating through vital eye technology or hemidiaphragm respiratory navigator on lung/liver interface.
	Flip angle:	Ernst angle for optimal SNR.	Contrast vs. SNR.	Ernst angle.

ECG, electrocardiogram; MRA: magnetic resonance angiography; SNR, signal to noise ratio.

### *How: Which Diameter to Report?*

Measurement of the aorta should ideally be performed in a 3D dataset using a double‐oblique angulation perpendicular to the vessel long‐axis.^1^, ^4^ With new automated software, double‐angulation is decreasingly time‐consuming and therefore is recommended whenever available as standard of care.^1^ Measurements can also be performed in standard 2D axial, coronal, and sagittal orientation, which have been shown to give an accurate assessment of aortic disease.^8^ A practical and efficient strategy can be to measure aortic diameters using axial, coronal, and sagittal orientations as first assessment before double‐oblique measurement of the maximal and minimal diameter for optimal measurement accuracy. If previous scans are available, a side‐by‐side comparison with the oldest scan is crucial to get the most sensitive comparison. For side‐by‐side comparison, measurement location is arbitrary as long as the same locations and techniques are used in both the new measurement as well as remeasurement of the previous scan. The used measurement technique and location should always be reported.

### *When: Timing in Cardiac Cycle?*

Echocardiography guidelines recommend that all aortic measurements except for the annulus should be performed during diastole.^9^ The European Society of Cardiology (ESC) guideline does not give specific recommendations; however, diastolic images give the best reproducibility because aortic pressure is the most stable and the proximal aorta shows less motion during diastole.^3^ In the American College of Cardiology / American Heart Association (ACC/AHA) and societal MRI guidelines, it is specified that electrocardiogram (ECG)‐gating should be performed at end‐diastole.^2^, ^7^ In conclusion, for the acquisition of the aorta using MRI it is advisable to use ECG‐gating triggered to end‐diastole, with an additional short stack of SSFP cines parallel to the valve through the left ventricular outflow tract for systolic measurement of the annulus (Fig. 1b) and end‐diastolic average cusp‐to‐commissure and largest cusp‐to‐cusp measurement of the sinus (Figs. 1c, 2c).

### *Where: Anatomical Landmarks*

**FIGURE 3:**
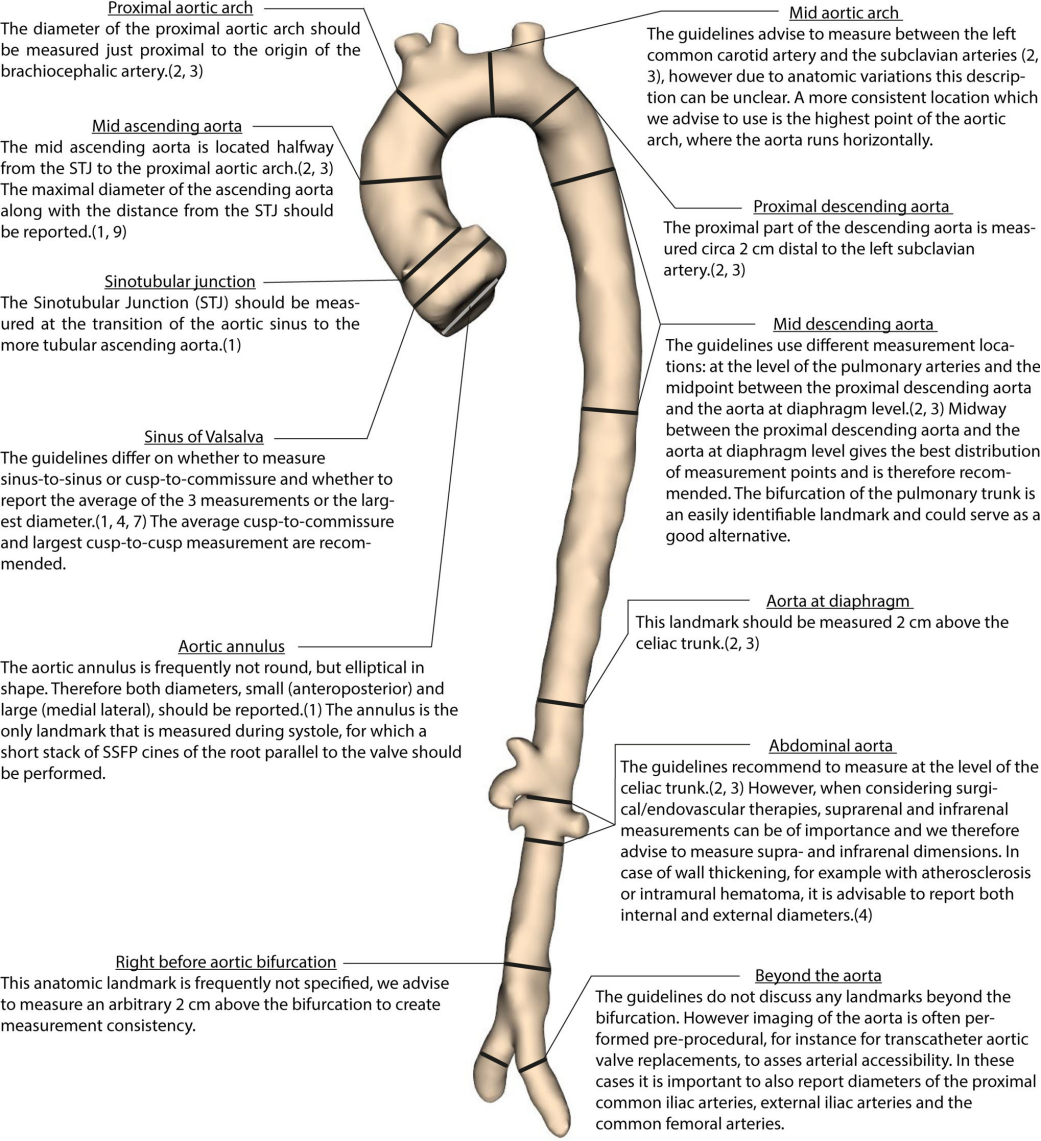
Recommended anatomical landmarks to measure the aorta.

Figure 3 shows the recommended anatomical landmarks to measure the aorta.

## Reference Values and Follow‐Up

MRI is ideal for follow‐up of aortic dimensions due to its capacity to image the entire aorta without using radiation or contrast. A normal reference range is imperative in the diagnosis and prognosis of aortic disease and in the timing of surgical interventions. MRI aortic reference values are available in a limited number of studies and differ in measurement and acquisition techniques, emphasizing the need for larger reference studies and updated guidelines.^10^ Although aortic diameters are highly correlated with body surface area,^1^ the guidelines still mainly use nonindexed diameters for timing of follow‐up and surgical intervention.^2^, ^3^ A short overview of follow‐up imaging frequency in aortic disease is shown in Table 2.

**TABLE 2:** Imaging Follow‐Up in Thoracic Aortic Disease^1^, ^2^, ^3^, ^8^

Clinical situation	Follow‐up
Aortic aneurysm	Aorta >40 mm: annual or biannual MRI/CT^*^ depending on aortic dilatation progression rate and family history
	Aorta >45 mm: annual or biannual MRI/CT^*^
Bicuspid valve	Normal aortic dimensions: MRI/CT^*^ every 3 to 5 years
	Aorta >40 mm: annual or biannual MRI/CT^*^ depending on aortic dilatation progression rate and family history
	Aorta >45 mm: annual or biannual MRI/CT^*^
Marfan’s syndrome	MRI at baseline and MRI/CT^*^ every 3 years;
	TTE annually if aortic diameter is stable <45 mm and negative family history of aortic dissection. >45 mm: annual or biannual MRI/CT^*^
Loeys‐Dietz syndrome	Annual MRI from brain to pelvis
Turner syndrome	Normal baseline measurement: MRI/CT/TTE^*^ every 5 to 10 years or preconception
Acute aortic dissection	TTE and MRI/CT^*^ at 1, 3, 6, and 12 months, then annual TTE and MRI/CT^*^
Chronic aortic dissection	TTE and MRI/CT^*^ every 2 to 3 years
IMH or PAU	MRI/CT^*^ at 1, 3, and 6 months, then annual MRI/CT*

* Selection of imaging modality for follow‐up is multifactorial, depending on imaging requirements, risks, and availability. It is desirable to use the same imaging modality over time to aid measurement consistency.

CT: computed tomography; IMH: intramural hematoma; MRI: magnetic resonance imaging; PAU: penetrating atherosclerotic ulcers; TTE: transthoracic echocardiography.

## Future Perspectives

**FIGURE 4:**
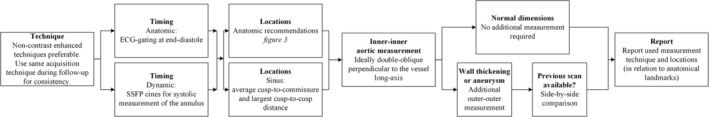
Flowchart summarizing the provided recommendations.

Better predictors of aortic dissection are needed, where the International Registry of the Aortic Dissection showed that >50% of dissections occurred at diameters below the cutoff for preemptive surgery.^11^ In this respect, MRI will take a predominant place in the diagnostic assessment of aortic pathology, where it is the only technique available to image the entire aorta with additional information on physical properties like distensibility, stiffness, wall shear stress, and blood flow patterns.^11^ Other geometric parameters like aortic length, cross‐sectional area, tortuosity, and volumetric measurements have been proposed as potentially more sensitive risk factors for aortic dissection.^11^ With the rise of artificial intelligence, it is expected that all these parameters can be generated with minimal or no input required. However, first a clear definition of how to measure the aorta is needed to create reliable input for deep‐learning training. Therefore, it is crucial to create uniformity by widely accepting MRI guidelines on how, when, and where to measure the aorta. The main recommendations provided in this article are summarized in a flowchart (Fig. 4).

## References

[1] Goldstein SA , Evangelista A , Abbara S , et al. Multimodality imaging of diseases of the thoracic aorta in adults: From the American Society of Echocardiography and the European Association of Cardiovascular Imaging: Endorsed by the Society of Cardiovascular Computed Tomography and Society for Cardiovascular Magnetic Resonance. J Am Soc Echocardiogr 2015;28(2):119‐182. 25623219 10.1016/j.echo.2014.11.015

[2] Hiratzka LF , Bakris GL , Beckman JA , et al. 2010 ACCF/AHA/AATS/ACR/ASA/SCA/SCAI/SIR/STS/SVM guidelines for the diagnosis and management of patients with thoracic aortic disease: A report of the American College of Cardiology Foundation/American Heart Association task force on practice guidelines, American Association for Thoracic Surgery, American College of Radiology, American Stroke Association, Society of Cardiovascular Anesthesiologists, Society for Cardiovascular Angiography and Interventions, Society of Interventional Radiology, Society of Thoracic Surgeons, and Society for Vascular Medicine. Circulation 2010;121(13):e266‐e369. 20233780 10.1161/CIR.0b013e3181d4739e

[3] Erbel R , Aboyans V , Boileau C , et al. 2014 ESC guidelines on the diagnosis and treatment of aortic diseases: Document covering acute and chronic aortic diseases of the thoracic and abdominal aorta of the adult. The task force for the diagnosis and treatment of aortic diseases of the European Society of Cardiology (ESC). Eur Heart J 2014;35(41):2873‐2926. 25173340 10.1093/eurheartj/ehu281

[4] Schulz‐Menger J , Bluemke DA , Bremerich J , et al. Standardized image interpretation and post‐processing in cardiovascular magnetic resonance — 2020 update: Society for Cardiovascular Magnetic Resonance (SCMR): Board of Trustees Task Force on standardized post‐processing. J Cardiovasc Magn Reson 2020;22(1):19. 32160925 10.1186/s12968-020-00610-6 PMC7066763

[5] Asch FM , Yuriditsky E , Prakash SK , et al. The need for standardized methods for measuring the aorta: Multimodality Core lab experience from the GenTAC registry. J Am Coll Cardiol Imaging 2016;9(3):219‐226. 10.1016/j.jcmg.2015.06.023 PMC4788536 26897684

[6] Li AE , Kamel I , Rando F , et al. Using MRI to assess aortic wall thickness in the multiethnic study of atherosclerosis: Distribution by race, sex, and age. AJR Am J Roentgenol 2004;182(3):593‐597. 14975953 10.2214/ajr.182.3.1820593

[7] Schulz‐Menger J , Bluemke DA , Bremerich J , et al. Standardized image interpretation and post processing in cardiovascular magnetic resonance: Society for Cardiovascular Magnetic Resonance (SCMR) board of trustees task force on standardized post processing. J Cardiovasc Magn Reson 2013;15:35. 23634753 10.1186/1532-429X-15-35 PMC3695769

[8] Mongeon FP , Marcotte F , Terrone DG . Multimodality noninvasive imaging of thoracic aortic aneurysms: Time to standardize? Can J Cardiol 2016;32(1):48‐59. 26724510 10.1016/j.cjca.2015.09.025

[9] Lang RM , Badano LP , Mor‐Avi V , et al. Recommendations for cardiac chamber quantification by echocardiography in adults: An update from the American Society of Echocardiography and the European Association of Cardiovascular Imaging. J Am Soc Echocardiogr 2015;28(1):1‐39.e14. 25559473 10.1016/j.echo.2014.10.003

[10] Kawel‐Boehm N , Maceira A , Valsangiacomo‐Buechel ER , et al. Normal values for cardiovascular magnetic resonance in adults and children. J Cardiovasc Magn Reson 2015;17:29. 25928314 10.1186/s12968-015-0111-7 PMC4403942

[11] Evangelista A . Imaging aortic aneurysmal disease. Heart (British Cardiac Society) 2014;100(12):909‐915. 24842834 10.1136/heartjnl-2013-305048

